# The Operative Treatment of Enlargement of the Prostate

**Published:** 1893-06

**Authors:** 


*The Operative Treatment of Enlargement of the Prostate.* By C. W. MANSELL MOULLIN. Pp. 82. London: JOHN BALE & SONS. 1892.

This is a series of lectures delivered by the author at the Royal College of Surgeons, and now published by him in book form, with some alterations and additions; the whole forming a very complete record of the views at present held on the surgical treatment of the prostate.

The author acknowledges throughout the obligation he is under to the late Mr. McGill of Leeds, whose work on this subject first drew attention to the favourable results attained by removal of prostatic overgrowths, although Mr. Mansell Moullin has discovered that Amussat performed the operation so far back as 1827, whereas McGill did his first operation in 1887. So great has been the favour bestowed upon the operation since McGill's writings, that no less than 95 operations are here tabulated, only 10 of which are prior to McGill's first case; while in a postscript the author acknowledges reports of many other unpublished cases, including 5 from Mr. Jordan Lloyd of Birmingham and 4 from Mr. Bruce Clarke.

The mortality after the supra-pubic operation is estimated at about 20 per cent., which is not great, considering the advanced age of most of the patients and the fatal nature of the malady. This mortality will, however, be doubtless diminished after further experience, and if the second half of the operations be reckoned separately, the mortality is even now only 15 per cent.

Full reference is made in the lectures before us to other operations upon the prostate besides McGill's, including especially the perineal operations still preferred by some, but most surgeons will agree with the author that McGill's supra-pubic method is the best yet devised.

